# Selected articles from the Fourth International Workshop on Semantics-Powered Data Mining and Analytics (SEPDA 2019)

**DOI:** 10.1186/s12911-020-01292-x

**Published:** 2020-12-14

**Authors:** Zhe He, Cui Tao, Jiang Bian, Rui Zhang

**Affiliations:** 1 School of Information, College of Communication and Information, Florida State University, 142 Collegiate Loop, Tallahassee, FL 32306-2100 USA; 2 School of Biomedical Informatics, University of Texas Health Science Center At Houston, Houston, TX USA; 3 Department of Health Outcomes and Biomedical Informatics, University of Florida, Gainesville, FL USA; 4 Institute for Health Informatics and College of Pharmacy, University of Minnesota, Minneapolis, MN USA

## Abstract

In this introduction, we first summarize the Fourth International Workshop on Semantics-Powered Data Mining and Analytics (SEPDA 2019) held on October 26, 2019 in conjunction with the 18th International Semantic Web Conference (ISWC 2019) in Auckland, New Zealand, and then briefly introduce seven research articles included in this supplement issue, covering the topics on Knowledge Graph, Ontology-Powered Analytics, and Deep Learning.

## Background

In the era of big data, the volume, the variety, as well as the velocity of data being generated have posed major challenges for people to leverage multiple data sets for decision making [1]. Ontologies and semantic standards have been widely used to tackle some of the challenges in big data analytics such as data integration and knowledge discovery [2]. In the biomedical domain, ontologies and controlled vocabularies are a cornerstone for health information systems including clinical decision support systems and electronic health record (EHR) systems [2, 3]. Moreover, rich vocabularies and semantic information embedded in the ontologies have been leveraged to extract clinically meaningful information from heterogenous data from various sources. In particular, they are instrumental in natural language processing and text mining [4]. As a notable example, the Unified Medical Language System, developed and maintained by the U.S. National Library of Medicine, has been widely used in informatics research and applications using data in social media, scientific literature, and EHRs [5]. Applications like PubMed, which uses the UMLS indirectly, has been used by millions of users worldwide for biomedical research.

The International Workshop on Semantics-Powered Data Mining and Analytics (SEPDA) has been established as an important venue for experts to discuss semantic-based methods and applications in health data analytics [6–8]. To continue our momentum, SEPDA 2019 was held on October 26, 2019, in conjunction with the 18th International Semantic Web Conference (ISWC 2019). Submissions were solicited on the topics including Semantics-Based Data Mining and Analytics, Ontologies and Controlled Vocabularies, Data Integration, and Applications. After the peer review by the program committee members, 11 papers were accepted for presentation and publication in the SEPDA 2019 workshop proceedings [9]. After the workshop, the authors of seven selected papers were invited to extend their workshop papers to journal papers by adding additional experiments and greater details of the methods, results, and discussion. Each of the extended papers was subsequently reviewed by two experts in the field followed by multiple rounds of revisions to ensure the highest scientific rigor and clear presentation.

In this editorial, we summarize the papers included in this supplement. We categorize them into three main themes: Knowledge Graph, Ontology-Powered Analytics, and Deep Learning.

## Knowledge graph

The majority of biomedical knowledge is still locked in text format such as those from textbook and scientific literature, while downstream applications such as those that provide clinical decision support still heavily rely on structured discrete data. Systems that curate knowledge graphs and knowledge bases from biomedical literature are rational intermediate steps. The paper from Rossanez et al. [10] introduced and evaluated a semi-automatic natural language processing (NLP) method that can generate knowledge graphs from biomedical texts. Their case study focused on Alzheimer’s disease and their evaluation results demonstrated reasonable performance of the ontology-linked knowledge graphs.

Deep learning, which can classify nodes in the knowledge graph with good predictive performance, suffers from poor interpretability. In the healthcare domain, interpretability of AI models is critical for clinical decision making. Vandewiele et al. [11] presented a new method called MINDWAL, an inherently interpretable technique for classifying nodes in a knowledge graph. This technique uses a recursive algorithm to induce multiple decision trees and then decouple the modeling with multiple using informative random walks, which will create high-dimensional binary features that can feed a classification algorithm. This model has an improved interpretability and a competitive performance in terms of accuracy compared to other baseline techniques (e.g., decision tree, random forest, transform + logistic regression, transform + random forest). This technique can be applied to knowledge graphs in the biomedical domain to classify nodes in the graph.

## Ontology-powered analytics

The needs to integrate diverse data sources across different domains (e.g., genetic factors and environmental exposures) and levels (e.g., individual traits as well as their interactions with the community) are growing so that a comprehensive examination of all potential risk factors is possible. The number of these multi-level integrative data analysis (mIDA) studies is increasing; nevertheless, the data integration processes in these mIDA studies are inconsistently performed and poorly documented. Zhang et al. [12] developed the ATTEST check list for standardized reporting of the variable and data source selection and subsequently the data integration processes. The novel piece of their study is the proposal to standardize the reports using an ontology, OD-ATTEST, that paves the way to enable sharing of mIDA study reports among researchers. Only when the selection and integration choices are clearly documented, the transparency and reproducibility of the studies can be warranted.

In [13], Zhang et al. proposed a semantic relationship mining method among disorders, genes, and drugs from different biomedical datasets. First, multiple heterogeneous biomedical datasets were converted and integrated into a resource description framework (RDF) storage system. Second, nine query patterns about genes, disorders, and drugs were presented. Third, the gene-disorder-drug semantic relationship mining algorithm was designed with these query patterns. The method was verified on SemMedDB, PharmGKB, KEGG, and Uniprot for Parkinson’s disease semantic relationship mining. The results demonstrated that the method has advantages in mining and integrating heterogeneous biomedical datasets.

Amith and colleagues utilized their dialogue ontology called the Patient Health Information Dialogue Ontology (PHIDO) [14] to control a software engine for dialogue management (“Conversational Ontology Operator”). Using utterance data collected from past Wizard of OZ simulations [15, 16], they described how their ontology-driven software engine could power various software agents to preform dialogue tasks from health-based counseling for the HPV vaccine [17]. Their paper also outlines a question-answering sub-system (“FOQUS”) that supplements the automated counseling of HPV vaccine where patients may ask questions. FOQUS utilizes a previous developed ontology knowledge base of HPV vaccine [18] to supply answers and was tested with question utterances from the aforementioned simulation. Their prototype engine presents some early showing of an ontology-based system to manage counseling methods for machines. Their future goal is to deploy this system to a live speech-enabled system to demonstrate its functional potential.

## Deep learning

Deep learning has transformed medicine in the past few years [19]. Predicting treatment effects based on patients’ personalized clinical status is vital in disease management. Traditional randomized controlled trials (RCT) usually are limited to a focused population and only evaluated the treatment effects after they have occurred [20]. EHRs containing large amounts of fine-grained clinical data provide a rich source to predict treatment effects. Chu et al. [21] proposed an adversarial deep treatment effect prediction (ADTEP) model based on auto-encoder and adversarial learning (AL). They encoded physical condition and treatment information for individual patients. An AL schema was also adopted to align the generated treatment with the actual performed treatments. The ADTEP model was evaluated on two clinical datasets and the results demonstrated its superiority compared with state-of-the-art methods.

Cancer survivors often experience emotional stress, post-traumatic stress disorder (PTSD), and other mental health issues. As such, they are at a high risk of self-destruction and harming others [22]. Early detection of mental health issues and early intervention would help prevent these undesired consequences. Social web such as Twitter allows people to share their experiences and opinions while keeping anonymous. Therefore, it is a great source for identifying cancer survivors with PTSD or other mental health issues. Ismail and colleagues [23] developed and evaluated a technique based on convolutional neural networks (CNN) to automatically classify tweets related to cancer survivors living with PTSD using word embeddings for text representation. The CNN-based model with word embeddings was trained to extract text features related to PTSD using a transfer learning approach and a depression lexicon. The results showed that the proposed model outperformed baselines including NBC, SVM, MLP, and CNN with n-grams for classifying the tweets.

## Discussion and conclusions

In this supplement of selected articles from the Fourth International Workshop on Semantics-Powered Data Mining and Analytics (SEPDA 2019), seven papers were accepted after a rigorous peer review process. These papers demonstrated the power of the semantic methods in various applications, many of which are addressing critical challenges in healthcare such as predicting treatment effect, identifying cancer survivors living with PTSD, and mining relationships among disorders, genes, and drugs from biomedical databases. We hope these papers will have sustainable impacts not only on biomedical and health informatics but also other related fields. We also hope more researchers will be motivated by these exciting results and join our effort to improve population health and advance biomedical research with semantics-powered data analytics over disparate datasets.

## Data Availability

Not applicable.
